# Bacteriological characteristics and changes of *Streptococcus pneumoniae* serotype 35B after vaccine implementation in Japan

**DOI:** 10.1017/S0950268824001031

**Published:** 2024-10-04

**Authors:** Haruko Miyazaki, Bin Chang, Michinaga Ogawa, Rie Shibuya, Misako Takata, Shigeki Nakamura, Kimiko Ubukata, Yoshitsugu Miyazaki, Tetsuya Matsumoto, Yukihiro Akeda

**Affiliations:** 1 Department of Microbiology, Tokyo Medical University, Tokyo, Japan; 2 Department of Bacteriology 1, National Institute of Infectious Diseases, Tokyo, Japan; 3 Department of Clinical Laboratory, Saiseikai Yokohamashi Tobu Hospital, Kanagawa, Japan; 4 Department of Fungal Infection, Leprosy Research Center, National Institute of Infectious Diseases, Tokyo, Japan; 5 Department of Infectious Diseases, International University of Health and Welfare, Chiba, Japan

**Keywords:** antibiotic resistance, bacterial typing, *Streptococcus pneumoniae* (pneumococcus), type 1 pilus, vaccination (immunization)

## Abstract

*Streptococcus pneumoniae* serotype 35B, a non-vaccine type, is a major contributor to the increase in pneumococcal infection post-vaccination. We aimed to understand the mechanism of its spread by characterizing 35B. The serotype, type 1 pilus (T1P) positivity, and antimicrobial susceptibility of 319 isolates in 2018–2022 were analysed and compared with those of isolates in 2014–2017 to find the changes. 35B accounted for 40 (12.5%) isolates. T1P positivity was notably higher in 35B (87.5%) than in the other serotypes. To confirm the role of T1P, an adhesion factor, we compared adherence to A549 cells between *T1P*-positive 35B isolates and their *T1P*-deficient mutants, showing contribution of T1P to adherence. Penicillin-non-susceptible rate of 35B was 87.5%, and meropenem-resistant 35B rate was 35.0%, which increased from 14.5% of 2014–2017 (*p* = 0.009). Multilocus sequence typing was performed in 35B strains. Prevalence of clonal complex 558, harbouring *T1P* and exhibiting multidrug non-susceptibility, suggested the advantages of 35B in attachment and survival in the host. The emergence of ST156 isolates, *T1P*-positive and non-susceptible to β-lactams, has raised concern about expansion in Japan. The increase of serotype 35B in pneumococcal diseases might have occurred due to its predominant colonizing ability after the elimination of the vaccine-serotypes.

## Introduction

*Streptococcus pneumoniae* is a commensal bacterium of the human upper respiratory tract and a major causative pathogen of diseases, such as meningitis, pneumonia, septicaemia, and otitis media. Pneumococcal vaccines effectively prevent pneumococcal infections; however, the increase of non-vaccine-type (NVT) infections after vaccine introduction is a concern [1–5]. In Japan, the 13-valent pneumococcal conjugate vaccine (PCV13: serotypes 1, 3, 4, 5, 6A, 6B, 7F, 9V, 14, 18C, 19F, 19A, and 23F) is currently routinely used for children, and the 23-valent pneumococcal polysaccharide vaccine (PPSV23: serotypes 1, 2, 3, 4, 5, 6B, 7F, 8, 9N, 9V, 10A, 11A, 12F, 14, 15B, 17F, 18C, 19F, 19A, 20, 22F, 23F, and 33F) is used for older adults. The prevalence of serotype 35B, an NVT, has increased in pneumococcal infections post-vaccination [4–6]. In our previous study of isolates primarily from sputum at an institution in Japan after the start of routine vaccination, 35B was the most common serotype, with 88.9% of the 35B isolates being penicillin non-susceptible [7]. This suggested that the predominance of 35B among the NVTs may arise from its superior colonization capability.

Type 1 pilus (T1P), an adhesion factor, may play a role in the establishment and virulence of *S. pneumoniae*, as shown in studies using the standard *S. pneumoniae* strain TIGR4 [8, 9]. In our prior study of isolates collected in 2014–2018, *T1P* was significantly more frequent (80.2%) in the 35B isolates than in the other serotypes [10], and multilocus sequence typing (MLST) revealed that clonal complex (CC) 558, which harbours *T1P*, constituted for 77.9% of 35B strains [10]. This suggested that T1P may be involved in the increase of 35B isolation rate. However, the role of T1P in adhesion of 35B has not been reported.

To elucidate the reason behind the spread of 35B, this study examined the serotype frequency, T1P positivity, and antimicrobial susceptibility, and clonal types of 35B clinical isolates collected in 2018**–**2022. We compared them to those in an earlier period, 2014–2017, to detect changes in the 35B isolates over the course of the 10-year implementation of the national vaccination programme. We also assessed the role of T1P in adherence by comparing adhesion rates to alveolar epithelial cells between T1P-positive clinical 35B isolates and their T1P-deficient mutants, to examine whether T1P contributes to adhesion of serotype 35B.

## Methods

### Isolates

We obtained 365 clinical isolates of *S. pneumoniae* during 2018–2022 from specimens, regardless of patient age or diagnosis, at Saiseikai Yokohamashi Tobu Hospital (Yokohama, Japan). In this study, if there were multiple isolates from the same patient within 3 months, the isolate from a sterile specimen or the first strain was selected. After these exclusions, 319 strains were used. For comparison, we used our data of 760 isolates in 2014–2017.

### T1P assay

T1P positivity was detected via PCR for the T1P subunit gene *rrgC* using primers from Regev-Yochay et al. (Supplementary Table S1) [11] and Quick Taq HS DyeMix (TOYOBO, Osaka, Japan). PCR was performed using a Veriti™ 96-well thermal cycler (Applied Biosystems, Foster City, CA, USA).

### Adherence experiments

Using two clinical isolates of 35B/ST558, namely SP212 and SP709, we constructed pneumococcal *T1P*-deficient mutants, SP212Δ*rrgABC* and SP709Δ*rrgABC* (Supplementary Table S2). Deletion of *rrgABC* was performed by crossover recombination as described previously [12]. Briefly, a km-pFW13 cassette with long flanking regions homologous to the target gene was generated via PCR using the primers listed in Supplementary Table S1. The PCR products were used to transform competent *S. pneumoniae* strains SP212 and SP709 [13]. Transformation was performed and deletion of *rrgABC* was confirmed via PCR using the primers listed in Supplementary Table S1.

Adherence assays were performed according to the method of Barocchi et al. [8], with some modifications. A549 alveolar epithelial cells (RIKEN Cell Bank, Tsukuba, Japan) were cultured in a 24-well plate at a density of 1 × 10^5^ cells/well in DMEM (ThermoFisher Scientific, Waltham, MA, USA) with 10% FBS for 2 days under 5% CO_2_. The bacteria were cultured for 16 h on blood agar medium, dissolved in DMEM to OD_600_ of 0.7, and 50 μL was added to A549 cells. The remaining bacterial solution was diluted and spread on blood agar medium and incubated for the colony count assay. The 24-well plate was incubated at 37°C under 5% CO_2_ for 30 min, washed twice with PBS before the addition of 200 μL 0.1% digitonin, and incubated at 37°C for 7 min. Subsequently, 800 μL of saline was added to each well. The solution was diluted and spread on blood agar medium and incubated for 20 h at 37°C under 5% CO_2_. Colonies were counted and the number of adherent or invading bacteria in each well calculated. The adherence rate was defined as the ratio of the number of bacteria adhering to the cells to the total number of bacteria added. The reduction rate was calculated as the difference in adherence rate between the parental strain and its *rrgABC*-deficient strain. Separately, bacteria that adhered to A549 cells were fixed, stained, and observed under a microscope (Olympus CKX41). Experiments were performed multiple times, independently.

### Antimicrobial susceptibility testing

Antimicrobial susceptibility testing was performed using the liquid microdilution method with the MICroFAST Panel Type7J (Beckman Coulter, Brea, CA, USA). The isolates were considered susceptible (S), intermediate (I), or resistant (R) according to Clinical & Laboratory Standards Institute (CLSI) criteria [14] for minimum inhibitory concentration (MIC). For penicillin (PEN), the breakpoints for oral administration (S: ≤0.06; I: 0.12–1; R: ≥2) were used. For ceftriaxone (CTRX), the breakpoints for meningitis (S: ≤0.5; I: 1; R: ≥2) were used. The relevant breakpoints were used for meropenem (MEPM) (S: ≤0.25; I: 0.5; R: ≥1), erythromycin (EM) (S: ≤0.25; I: 0.5; R: ≥1), clindamycin (CLDM) (S: ≤0.25; I: 0.5; R: ≥1), levofloxacin (LVFX) (S: ≤2; I: 4; R: ≥8), and vancomycin (VCM) (S: ≤1). For minocycline (MINO), the breakpoints for tetracycline from the CLSI M100 Ed33 (2023) standards were used (S: ≤1; I: 2; R: ≥4), as CLSI does not specify breakpoints for MINO.

### Serotyping and multilocus sequence typing

Isolates were cultured on 5% blood agar plates at 37°C in a 5% CO2 environment. DNA was extracted using a Cica Geneus DNA Extraction Reagent (Kanto Chemical Co., Tokyo, Japan) according to the manufacturer’s instructions. Capsular serotypes were identified via sequential multiplex PCR [15] using a QIAGEN Multiplex PCR Kit (QIAGEN, Hilden, Germany) or via the Quellung reaction with serotype-specific rabbit antisera (Statens Serum Institut, Copenhagen, Denmark). We defined 11A/11D because PCR and Quellung reaction cannot distinguish between 11A and 11D [16]. Strains for which the serotype could not be determined via PCR or Quellung reaction were defined as non-typeable. Isolates were classified as vaccine type (VT), which includes PCV13 or PPSV23 serotypes and serotype 6C (cross-immunogenic with 6A and affected by PCV13), or as NVT, not covered by PCV13 and PPSV23. Sequence type (ST) of the *S. pneumoniae* isolates was determined based on the sequences of seven housekeeping genes (*aroE*, *gdh*, *gki*, *recP*, *spi*, *xpt*, and *ddl*) [17]. Allelic numbers and STs for 123 *S. pneumoniae* 35B isolates were assigned based on information from the pneumococcal MLST website [18]. CCs were determined according to the definition of eBURST [16]. Strains for which five or more of the seven alleles were identical were classified as belonging to a CC.

### Statistical analysis

Statistical analysis was performed using 2 × 2 chi-square test and Fisher’s exact test. Adherence experiment was evaluated using Student’s *t*-tests. Statistical significance was set at *p* < 0.05.

### Ethics statement

This study was approved by the ethics committees of Saiseikai Yokohamashi Tobu Hospital (Approval No. 2016002) and Tokyo Medical University (Approval No. 2016-218). Patient data were anonymized for the analysis.

## Results

### Origin of isolates and serotype distribution

Table 1 presents the ages of the individuals from whom the strains were isolated and the specimen distribution. Isolates from individuals <15-years old were more common than those from individuals ≥15-years old. The proportion of isolates from individuals ≥15-years old was higher in 2018–2022 than in 2014–2017. Together, sputum, bronchial, and nasopharyngeal samples accounted for 95.7% of the specimens in 2014–2017 and 94.0% in 2018–2022. The frequency of isolates from sterile specimens (blood, cerebrospinal fluid, and pleural fluid) was 3.4% in 2014–2017 and 5.0% in 2018–2022. No significant differences were observed between two periods in proportions of specimens of each origin, except for nasopharyngeal samples.

**Table 1.** Patient’s age and sample of isolated pneumococcal strains

	2014–2017	2018–2022	
	*n* (%)	*n* (%)	*p*-value
Age			
<15 years	478 (62.9)	177 (55.5)	**0.023**
≥15 years	282 (37.1)	142 (44.5)	
Sample			
Sputum	619 (81.4)	274 (85.9)	0.078
Bronchial sample	55 (7.2)	16 (5.0)	0.179
Nasopharyngeal sample	53 (7.0)	10 (3.1)	**0.014**
Blood	21 (2.8)	15 (4.7)	0.106
Otorrhea	4 (0.5)	2 (0.6)	0.806
Cerebrospinal fluid	3 (0.4)	1 (0.3)	0.728
Pleural fluid	2 (0.3)	0 (0)	0.887
Others	3 (0.4)	1 (0.3)	0.728
Total	760	319	

p-values less than 0.05 were shown in bold.

**Figure 1.**
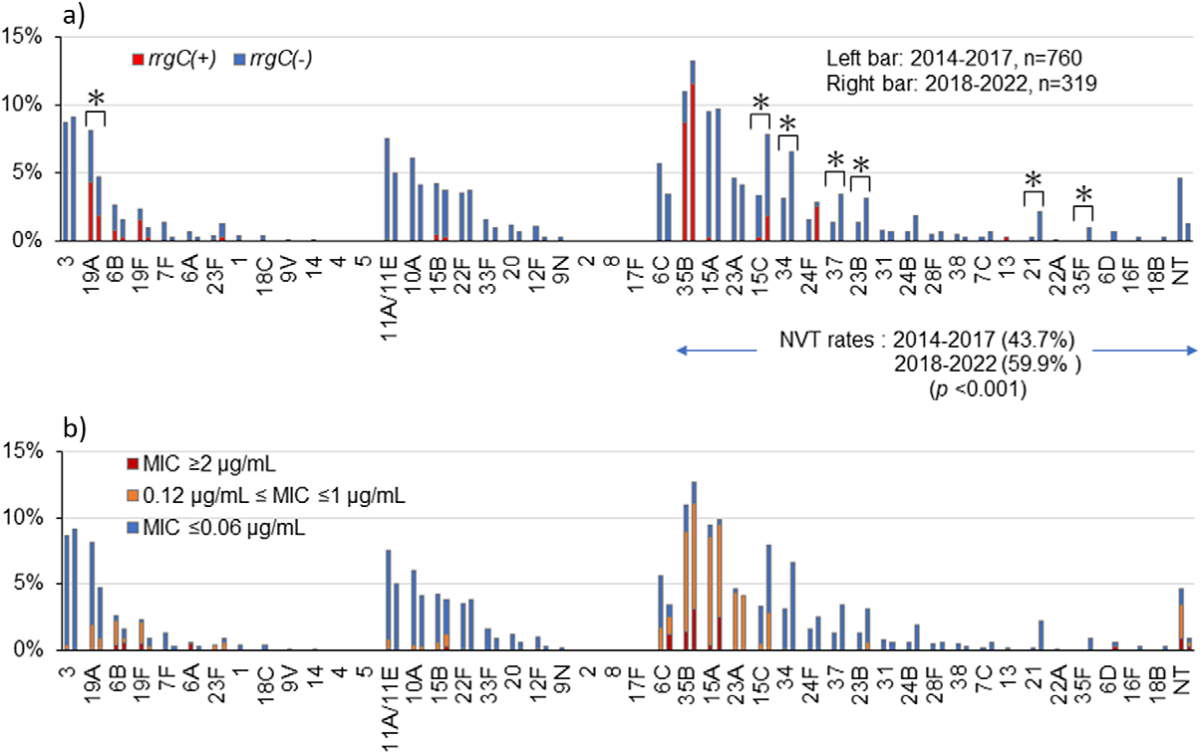
Serotype distribution of the pneumococcal isolates. For each serotype, the left bar shows its frequency in 2014–2017 (760 isolates in total), and the right bar shows its frequency in 2018–2022 (319 isolates in total). (a) Serotype and *T1P* positivity. *p < 0.05, comparing serotype frequencies between the periods. NVT: serotypes other than PCV13, PPSV23, or serotype 6C. (b) Serotype and penicillin susceptibility. MIC: minimum inhibitory concentration (μg/mL).

Figure 1 shows the serotype distribution in the two periods. The proportion of NVT strains significantly increased from 43.7% in 2014–2017 to 59.9% in 2018–2022 (*p* < 0.001) (Figure 1a). Serotype 35B accounted for 83 (10.9%) of 760 *S. pneumoniae* isolates in 2014–2017 and 40 (12.5%) of 319 isolates in 2018–2022; it was the most frequent serotype in both periods, followed by serotype 15A. The frequency of serotype 19A was significantly decreased, whereas that of serotypes 15C, 34, 37, 23B, 21, and 35F were significantly increased (Figure 1a).

### Serotype and T1P positivity

Figure 1a and Supplementary Table S3 show the relationship between serotype and *T1P* positivity among the isolates in this study. The *T1P* prevalence in NVT was 21.7% in 2014–2017 and 25.7% in 2018–2022, which were significantly higher than those in VT (13.6% and 7.8%, respectively) (*p* = 0.003 and *p* < 0.001, respectively). For serotype 35B, *T1P* was present in 79.5% in 2014–2017 and 87.5% in 2018–2022, which was significantly more frequent than that of other comparable serotypes, except for 19F, 13, and 24F. Although *T1P*-harbouring 35B strains were the most common in NVT, notably, *T1P* positive rates of 15C and 24F increased, and the isolation frequency of these serotypes also increased. In particular, eight of the nine isolates of 24F were *T1P-*positive in 2018–2022, whereas all 24F isolates in 2014–2017 were *T1P*-negative.

### Contribution of T1P in pneumococcal adhesion to A549 cells

**Figure 2.**
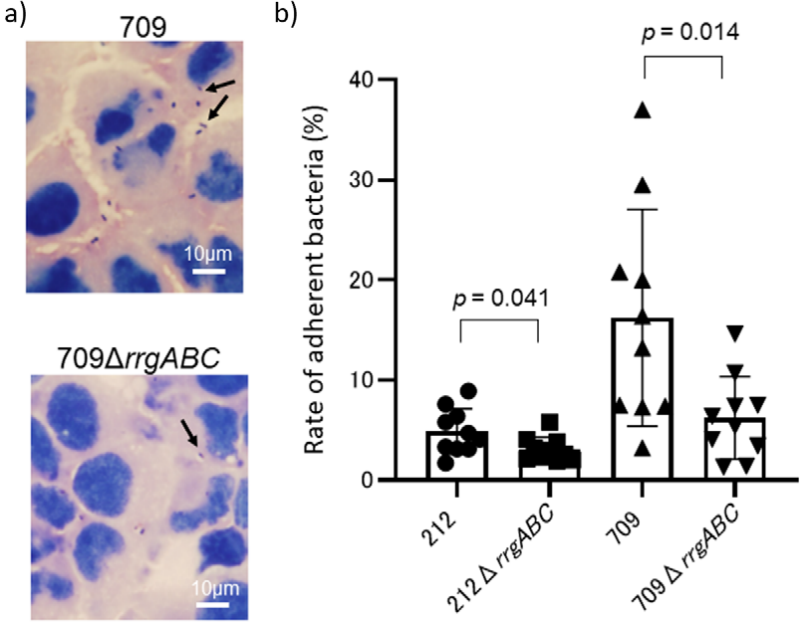
Adherence of piliated *Streptococcus pneumoniae* serotype 35B strains and their type 1 pilus-deficient mutants to A549 cells, 30 min after addition of the bacteria. (a) Microscopic images of SP709 and its type 1 pilus-deficient SP709Δ*rrgABC* strains. Bacteria were visualized using Gram staining. Arrows indicate pneumococci. (b) Adherence rates of SP212, SP212Δ*rrgABC*, SP709, and SP709Δ*rrgABC.* For comparing, the adherence rate was defined as the ratio of the number of bacteria adhering to the cells to the number of bacteria added. Experiments were performed multiple times, independently.

Figure 2a shows Gram stained microscopic images of adherent SP709 and its *T1P*-deficient strain SP709Δ*rrgABC* to A549 cells. Figure 2b shows the adherence rates of SP212, SP709, and their respective *T1P*-deficient strains to A549 cells. The adhesion rates were lower in the *T1P*-deficient strains than in their parental strains (reduction rate for SP212: 1.8 ± 0.021%, *p* = 0.041, and SP709: 10.0 ± 0.093%, *p* = 0.014), reconfirming the contribution of T1P to adherence to host cells.

### Serotype and antimicrobial susceptibility

**Figure 3.**
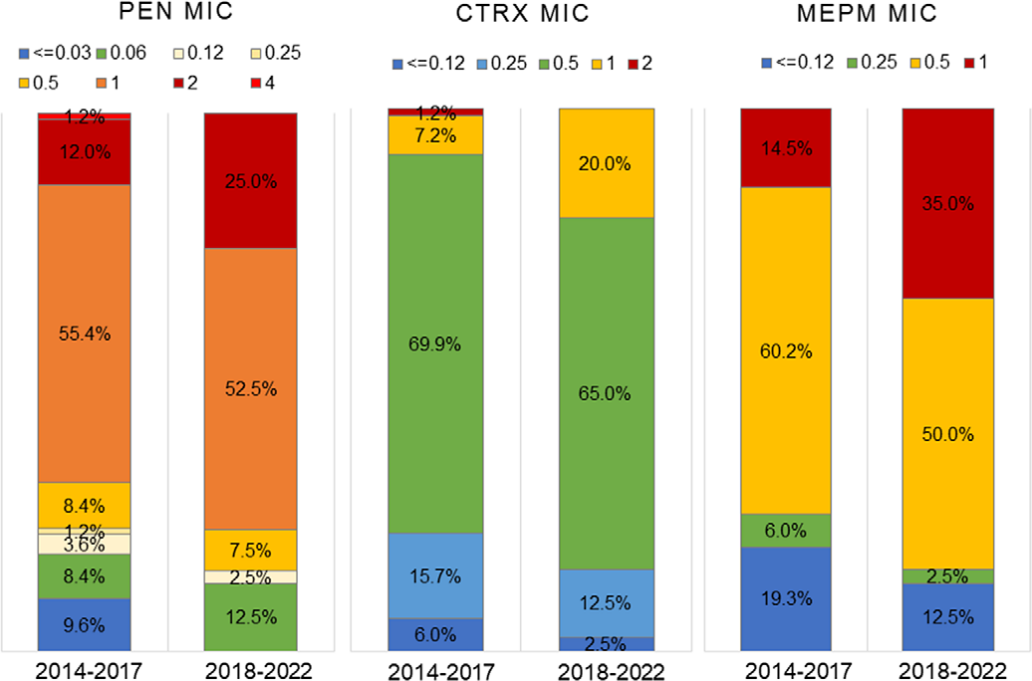
Susceptibility of *Streptococcus pneumoniae* serotype 35B isolates to β-lactams. MIC: minimum inhibitory concentration (μg/mL); PEN: penicillin; CTRX: ceftriaxone; MEPM: meropenem. The number of isolates was 83 in 2014–2017 and 40 in 2018–2022.

Figure 1b and Supplementary Table S4 show the serotypes and PEN susceptibilities of the strains. Non-susceptible strains (MIC ≥0.12) accounted for 37.5% in 2014–2017 and 36.1% in 2018–2022 among all strains, with no significant difference between the two periods. However, PEN–non-susceptible strains were significantly more frequent among NVT (59.6% in 2014–2017 and 48.7% in 2018–2022) than among VT (20.3% and 17.3%, respectively) (both periods: *p* < 0.001). The most common serotype among PEN–non-susceptible strains in both periods was 35B, followed by 15A and 23A. The percentage of strains with PEN-resistant (MIC ≥2.0) in all isolates significantly increased from 4.5% in 2014–2017 to 8.5% in 2018–2022 (*p* = 0.009). Particularly for serotype 15A, the percentage of PEN-resistant strains significantly increased (*p* = 0.003). Serotype 35B accounted for 32.4% of the PEN-resistant isolates in 2014–2017 and 37.0% in 2018–2022 and was the most frequent serotype. The distribution of MICs for PEN, CTRX, and MEPM among the 35B isolates is shown in Figure 3. The rates of PEN–non-susceptible 35B isolates were 81.9% and 87.5%, whereas CTRX–non-susceptible (MIC ≥1) isolates were 8.4% and 20.0%, and MEPM–non-susceptible (MIC ≥0.5) isolates were 74.7% and 85.0% in 2014–2017 and 2018–2022, respectively. The differences in these rates between the two periods were not statistically significant. Notably, the rate of MEPM-resistant (MIC = 1) 35B strains significantly increased from 14.5% to 35.0% (*p* = 0.009).

### Distribution of 35B sequence types and its relationship with T1P positivity and antimicrobial susceptibility

**Figure 4.**
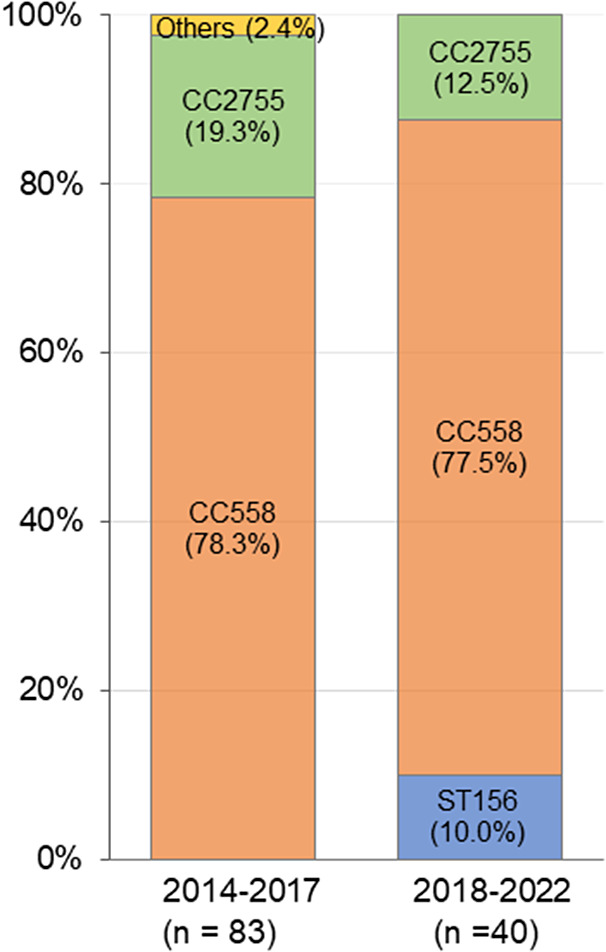
Clonal distribution of *Streptococcus pneumoniae* serotype 35B isolates in 2014–2017 and 2018–2022. ST: sequence type; CC: clonal complex.

Figure 4 shows the ST distribution of 35B isolates. Overall, 63 of 83 (78.3%) 35B strains in 2014–2017 and 31 of 40 (77.5%) strains in 2018–2022 were CC558. The second most common type was CC2755 (19.3% at 2014–2017 and 12.5% at 2018–2022). Notably, four strains (10.0%) in 2018–2022 were ST156, which was not detected in 2014–2017. Table 2 shows *T1P* positivity and antimicrobial susceptibility against PEN, CTRX, MEPM, EM, CLDM, LVFX, MINO, and VCM for each CC of 35B strains. All CC558 strains in 2018–2022 were *T1P*-positive and non-susceptible to PEN and EM. Further, 30 of 31 (96.8%) strains of CC558 were non-susceptible to MINO, of which two were also resistant to CLDM and one was resistant to LVFX. MEPM-resistant strains in CC558 showed an increasing trend – from 18.5% to 35.5% (*p* = 0.068). All the CC2755 isolates were T1P-negative. In 2018–2022, all five CC2755 strains were PEN-susceptible but resistant to EM and MINO, and four of which were also resistant to CLDM (Table 2). All four ST156 isolates were *T1P*-positive and non-susceptible to PEN, MEPM, and EM, and one was also non-susceptible to MINO. In contrast to CC558, 93.5% of which had MIC ≥4 for MINO, all ST156 strains had MIC ≤2 for MINO (Table 2). The rate of strains with MIC ≥4 for MINO among all 35B isolates was significantly increased (Table 2).

**Table 2.** Type 1 pilus and antimicrobial susceptibilities in sequence types of 35B isolates

		CC558		CC2755		ST156		Others		Total		
		2014–2017	2018–2022	2014–2017	2018–2022	2014–2017	2018–2022	2014–2017	2018–2022	2014–2017	2018–2022	*p*-value
		*n* (%)	*n* (%)	*n* (%)	*n* (%)	*n* (%)	*n* (%)	*n* (%)	*n* (%)	*n* (%)	*n* (%)	
Isolates		65	31	16	5	0	4	2	0	83	40	
T1P:	+	65 (100)	31 (100)				4 (100)	1 (50.0)		66 (79.5)	35 (87.5)	0.406
	−			16 (100)	5 (100)			1 (50.0)		17 (20.5)	5 (12.5)	
PEN:	R	11 (16.9)	7 (22.6)				3 (75.0)			11 (13.3)	10 (25.0)	0.105
	I	54 (83.1)	24 (77.4)	3 (18.8)			1 (25.0)			57 (68.7)	25 (62.5)	0.496
	S			13 (81.3)	5 (100)			2 (100)		15 (18.1)	5 (12.5)	0.600
CTRX:	R	1 (1.5)								1 (1.2)		0.708
	I	6 (9.2)	5 (16.1)				3 (75.0)			6 (7.2)	8 (20.0)	0.074
	S	58 (89.2)	26 (83.9)	16 (100)	5 (100)		1 (25.0)	2 (100)		76 (91.6)	32 (80.0)	0.123
MEPM:	R	12 (18.5)	11 (35.5)				3 (75.0)			12 (14.5)	14 (35.0)	**0.009**
	I	50 (76.9)	19 (61.3)				1 (25.0)			50 (60.2)	20 (50.0)	0.282
	S	3 (4.6)	1 (3.2)	16 (100)	5 (100)			2 (100)		21 (25.3)	6 (15.0)	0.289
EM:	R	60 (92.3)	30 (96.8)	13 (81.3)	5 (100)		4 (100)	2 (100)		75 (90.4)	39 (97.5)	0.292
	I	3 (4.6)	1 (3.2)							3 (3.6)	1 (2.5)	0.829
	S	2 (3.1)		3 (18.6)						5 (6.0)		0.272
CLDM:	R	8 (12.3)	2 (6.5)	11 (68.8)	4 (80.0)			2 (100)		21 (25.3)	6 (15.0)	0.289
	I	1 (1.5)								1 (1.2)		0.708
	S	56 (86.2)	29 (93.5)	5 (31.3)	1 (20.0)		4 (100)			61 (73.5)	34 (85.0)	0.232
LVFX:	R		1 (3.2)								1 (2.5)	0.708
	S	65 (100)	30 (96.8)	16 (100)	5 (100)		4 (100)	2 (100)		83 (100)	39 (97.5)	
MINO:	R	46 (70.8)	29 (93.5)	4 (25.0)	5 (100)					50 (60.2)	34 (85.0)	**0.011**
	I	9 (13.8)	1 (3.2)	8 (50.0)			1 (25.0)	1 (50.0)		18 (21.7)	2 (5.0)	**0.037**
	S	10 (15.4)	1 (3.2)	4 (25.0)			3 (75.0)	1 (50.0)		15 (18.1)	4 (10.0)	0.371
VCM:	S	65 (100)	31 (100)	16 (100)	5 (100)		4 (100)	2 (100)		83 (100)	40 (100)	
PEN, EM, MINO: R/I		57 (87.7)	30 (96.8)	2 (12.5)			1 (25.0)			59 (71.1)	31 (77.5)	0.593
PEN, EM, CLDM, MINO: R/I		9 (13.8)	2 (6.5)	1 (6.3)						10 (12.0)	2 (5.0)	0.363
PEN, EM, MINO, LVFX: R/I			1 (3.2)								1 (2.5)	0.708
PEN: S; EM, CLDM, MINO: R/I				10 (62.5)	4 (80.0)			1 (50.0)		11 (13.3)	4 (10.0)	0.824

*Note*: CC558: 73 of ST558 and 23 of non-ST558 isolates; CC2755: 17 of ST2755 and 4 of non-ST2755 isolates; T1P: type 1 pilus gene; +: T1P positive; −: T1P negative; PEN: penicillin; CTRX: ceftriaxone; MEPM: meropenem; EM: erythromycin; CLDM: clindamycin; LVFX: levofloxacin; MINO: minocycline; VCM: vancomycin; R: resistant; I: intermediate; S: susceptible. Breakpoints for PEN (S: ≤0.06; I: 0.12–1; R: ≥2), CTRX (S: ≤0.5; I: 1; R: ≥2), MEPM (S: ≤0.25; I: 0.5; R: ≥1), EM (S: ≤0.25; I: 0.5; R: ≥1), CLDM (S: ≤0.25; I: 0.5; R: ≥1), LVFX (S: ≤2; I: 4; R: ≥8), MINO (S: ≤1; I: 2; R: ≥4), and VCM (S: ≤1) were used.

*p*-values less than 0.05 were shown in bold.

## Discussion

The characteristics and their changes of serotype 35B isolates, which has increased burden after routine vaccination introduced, were investigated. We showed the contribution of T1P, of which 35B had higher positive rate than the other serotypes, in adhesion to host cells. The antimicrobial susceptibility and sequence types among 35B were changed in 10 years after vaccine introduction. These findings will provide useful information for predictions and countermeasures of pneumococcal infection.

In Japan, PCV13 became a routine vaccination for children in 2013, and PPSV23 for the older population in 2014. Although these vaccines reduced the isolation rate of VT pneumococci, the vaccine pressure caused changes in serotype distribution of isolates. As we collected isolates from clinical specimens regardless of confirmed diagnosis, the number of strains isolated from patients with invasive pneumococcal diseases (IPD) was 42 (3.8%), and most strains in this study might have been non-invasive or colonized. Therefore, the spread of serotypes among the population in this region, regardless of whether they cause infections, could be investigated. In our previous study, 35B was the most commonly isolated serotype in 2014–2016 [7], even though those were not from IPD. Two 35B strains derived from IPD have been isolated since 2019 in our collection. In the national IPD surveillance for adults from 2014, 35B used to be a small proportion of all IPD isolates [16]; however, it has increased annually and became the most common serotype in 2022 [19]. This change follows past trends in the United States, with earlier vaccine introductions than in Japan [20, 21]. Although serotype 35B is typically considered less virulent and rarely causes IPD, we suggest that it has established widely in place of VT strains following vaccine implementation, leading to its increased frequency in IPD isolates, especially from the elderly or individuals with risk, because pneumococcal diseases begin with colonization. Therefore, by examining regional spread of serotypes, including carriage, we can predict serotypes that may cause infections, including IPD, in the future.

We previously reported that 35B had more strains with T1P than other serotypes, and the majority of ST harbouring *T1P* was CC558 [10]. A previous study using *S. pneumoniae* TIGR4 (serotype 4) showed that T1P is involved in the adhesion to lung epithelial cells and pathogenicity in mice [8]. Therefore, we performed the experiment using our clinical 35B isolates and their *T1P*-deficient mutants to confirm the role of T1P in adhesion of serotype 35B. The adherence rates of the *T1P*-deficient strains were significantly lower than those of the parental strains, suggesting that T1P contributes to host cell attachment even in 35B. Pili on the surface beyond the capsular membrane may be one of the first molecules to act in adherence. However, *T1P*-deficient strains did not completely lose adherence, and the degree of adherence differed between isolates. Considering some other adhesion factors in *S. pneumoniae* reported previously [22], it is unclear to what extent T1P contributes to the increase in colonization of 35B. Moreover, most isolates of serotype 15A, the second most common NVT, were T1P-negative. We performed adherence experiment using some serotypes of *T1P*-negative and observed that the adherence rates were strain-dependent. Even a 35B/ST2755, strain of natively *T1P*-negative, exhibited higher adherence rates than 35B/ST558 strains of *T1P*-positive (data not shown). These suggested that T1P is just one of the adhesion factors and contribute to enhance adherence in *S. pneumoniae* colonization. Further studies are needed to identify other important and critical reasons behind the spread of 35B. Serotype 15C, which showed an increased rate of *T1P*-positive isolates, and serotype 24F, which replaced *T1P*-negative isolates with *T1P*-positive isolates, is of great interest. Notably, 15C and 24F showed an increased proportion of isolates, suggesting the possible involvement of *T1P* in colonization (Figure 1a). *T1P*-positive strains may emerge and proliferate in other NVTs as well. Furthermore, serotype 19A, which rapidly increased after the introduction of seven-valent pneumococcal conjugate vaccine [23], was a serotype with a high frequency of T1P-positive strains [10]; this serotype, included in PCV13, decreased after PCV13 introduction (Figure 1a). The increase in the frequency of 35B post-PCV13 suggests a potential link to T1P presence. Given that *T1P* expression in TIGR4 has been reported to be biphasic [24], further studies are needed to elucidate *T1P* expression in the host and factors stimulating its expression.

Serotype 35B had a high rate of multidrug–non-susceptible strains and showed an increase of MEPM-resistant rate. The trend towards increased MICs for β-lactams must be advantageous for colonization of 35B. In serotype 35B, T1P-positive strains were highly PEN-non-susceptible. However, since the T1P-positive 24F strains were PEN-susceptible, and since our antimicrobial susceptibility test using the *T1P*-deficient ST558 mutants showed no change in PEN MIC with the parental strains (data not shown), we believe that T1P does not contribute to PEN-non-susceptibility. Serotypes 15A and 23A in NVT also exhibited high rates of PEN–non-susceptible strains and were prevalent behind 35B. It is hypothesized that the selective pressure exerted by using β-lactams allows low-susceptible bacteria colonizing the nasopharyngeal cavity to survive without being eliminated, and exposure to β-lactams further increases resistance and facilitates colonization. The increased MICs to β-lactams in 35B in this study suggested that 35B may be conferred additional genetic advantage in retention. Li et al. [25] reported a correlation between β-lactam MICs and penicillin binding protein (PBP) types of *S. pneumoniae.* Investigating PBP types in strains collected in local areas will be useful for characterizing *S. pneumoniae* distribution and providing information for the prevention and treatment of pneumococcal infections. The frequency of MINO-resistant 35B isolates will depend on the distribution of ST in future.

Although ST558 was the predominant clone among 35B strains in our study even in 2014–2017, national IPD surveillance in Japan from 2010 to 2017 indicated that ST2755 was more prevalent than ST558 among 35B isolates [3]. ST2755 is a unique clone found in Japan and the surrounding countries. In the United States, the common clone of 35B was CC452, ahead of ST558 [26]. The frequency of non-piliated and PEN-susceptible ST2755 strains in Japan is declining, similar to that of CC452 strains in the United States [26]. Alternatively, ST156 emerged in our collection at 2018–2022, accounting for 4 of the 40 isolates of 35B. 35B/ST156 was reported to be caused by capsular switching between 35B/ST558 and 9V/ST156 [20] and increased after PCV13 introduction in the United States [27]. It should be noted that ST156 is *T1P*-positive and PEN–non-susceptible. Although ST156 was only one of 106 isolates of 35B in the national IPD surveillance in Japan from 2010 to 2017 [3] and no 35B/ST156 was found in another nationwide surveillance in 2012–2017 [28], we need to continue to monitor whether its frequency will increase in Japan. PCV15 was introduced in 2023 in Japan and PCV20 is scheduled for introduction in 2024; however, serotype 35B is not included. PCV21, which is currently under consideration, includes 35B, and is expected to provide additional protection against pneumococcal infections.

This study had some limitations. First, being conducted in a single central hospital, there are concerns regarding geographical variation, and the representativeness of our data for all regions of Japan is uncertain. Second, as collection began after initiation of routine vaccinations, we could not conduct comparisons with pre-vaccination isolates from this region. Third, the extent to which T1P and antimicrobial susceptibility contribute to the increased rate of 35B remains unclear, and there must be other factors of significance for its distribution to be studied.

In conclusion, we speculated that after the elimination of the vaccine-type serotypes, *S. pneumoniae* serotype 35B was prevalent owing to its predominant colonizing ability, which may be enhanced by presence of type 1 pilus and by its low antimicrobial susceptibility. Continued investigation of serotype distribution, *T1P* positivity, antimicrobial susceptibility, and sequence type of isolates including colonized strains will provide important information for the future treatment and prevention of pneumococcal infections in Japan.

## Supporting information

Miyazaki et al. supplementary material 1 Miyazaki et al. supplementary material

Miyazaki et al. supplementary material 2 Miyazaki et al. supplementary material

Miyazaki et al. supplementary material 3 Miyazaki et al. supplementary material

Miyazaki et al. supplementary material 4 Miyazaki et al. supplementary material

## Data Availability

The data are available upon request at hmiya@tokyo-med.ac.jp.

## Supplementary material

The supplementary material for this article can be found at http://doi.org/10.1017/S0950268824001031.
