# Supplementary material for: Bacteriological characteristics and changes of Streptococcus pneumoniae serotype 35B after vaccine implementation in Japan
Source: Epidemiol Infect. 2024 Oct 4;152:e114. doi: 10.1017/S0950268824001031 (PMC11450500; doi:10.1017/S0950268824001031)
Supplement: Miyazaki et al. supplementary material 1 — Miyazaki et al. supplementary material [file S0950268824001031sup001.docx]

Supplementary Table S1.　Primer sequences

| primer | sequence | Source |
| --- | --- | --- |
| *rrgC*-F | GCTCTGTGTTTTTCTCTTGTATGG | Regev-Yochay G, et al. Vaccine 28: 4842-4846, 2010 |
| *rrgC*-R | ATCAATCCGTGGTCGCTTGTTATTTTTA | Regev-Yochay G, et al. Vaccine 28: 4842-4846, 2010 |
| *rrgABC*-NF | TCCCGACGTTTCCAAGTGAG | This study |
| *rrgABC*-NR | CGTACGCTAGCAATTGTACATTATCCTGCA  ACTGCCTTCTGAA | This study |
| *rrgABC*-CF | AATGGACTAATGAAAATGTAAATTTAACTA CAAGCGACCACGGATTGATGTGCC | This study |
| *rrgABC*-CR | GCGCAAGCGTCGAATAATCC | This study |
| Check primer-F | GGACTCAGGGAACTCAAGTGG | This study |
| Check primer-R | GATCTAGAGCTCGAGGATCC | This study |
