# Supplementary material for: Bacteriological characteristics and changes of Streptococcus pneumoniae serotype 35B after vaccine implementation in Japan
Source: Epidemiol Infect. 2024 Oct 4;152:e114. doi: 10.1017/S0950268824001031 (PMC11450500; doi:10.1017/S0950268824001031)
Supplement: Miyazaki et al. supplementary material 2 — Miyazaki et al. supplementary material [file S0950268824001031sup002.docx]

Supplementary Table S2. 　Strains used for adherence experiment

| Strain | Relevant characteristics | Source |
| --- | --- | --- |
| *Streptococcus pneumoniae* SP212 | Clinical isolate of type 35B/ST558 | This study |
| *Streptococcus pneumoniae* SP709 | Clinical isolate of type 35B/ST558 | This study |
| SP212Δ*rrgABC* | rrgABC::km-pFW13 (aphD) | This study |
| SP709Δ*rrgABC* | rrgABC::km-pFW13 (aphD) | This study |
