# Supplementary material for: Bacteriological characteristics and changes of Streptococcus pneumoniae serotype 35B after vaccine implementation in Japan
Source: Epidemiol Infect. 2024 Oct 4;152:e114. doi: 10.1017/S0950268824001031 (PMC11450500; doi:10.1017/S0950268824001031)
Supplement: Miyazaki et al. supplementary material 3 — Miyazaki et al. supplementary material [file S0950268824001031sup003.docx]

Supplementary Table S3. Serotype and T1P positive rate of *Streptococcus pneumoniae* isolates

|  |  | Isolates | | | T1P (+) | | |
| --- | --- | --- | --- | --- | --- | --- | --- |
|  |  | 2014-2017 | 2018-2022 | *p* | 2014-2017 | 2018-2022 | *p* |
|  |  | ｎ (%^c^) | ｎ (%^c^) |  | n (%^d^) | n (%^d^) |  |
| VT^a^ | | 428 (56.3) | 128 (40.1) | **<0.001** | 58 (13.6) | 10 (7.8) | 0.082 |
|  | 3 | 66 (8.7) | 29 (9.1) | 0.830 |  |  |  |
|  | 19A | 62 (8.2) | 15 (4.7) | **0.044** | 33 (53.2) | 6 (40.0) | 0.528 |
|  | 6B | 20 (2.6) | 5 (1.6) | 0.402 | 6 (30.0) | 1 (20.0) | 1.000 |
|  | 19F | 18 (2.4) | 3 (0.9) | 0.191 | 12 (66.7) | 1 (33.3) | 0.531 |
|  | 7F | 10 (1.3) | 1 (0.3) | 0.245 |  |  |  |
|  | 6A | 5 (0.7) | 1 (0.3) | 0.806 | 1 (20.0) |  | 1.000 |
|  | 23F | 3 (0.4) | 4 (1.3) | 0.235 | 1 (33.3) | 1 (25.0) | 1.000 |
|  | 1 | 3 (0.4) |  | 0.624 |  |  |  |
|  | 18C | 3 (0.4) |  | 0.624 |  |  |  |
|  | 9V | 1 (0.1) |  | 0.654 |  |  |  |
|  | 14 | 1 (0.1) |  | 0.654 |  |  |  |
|  | 11A/E | 57 (7.5) | 16 (5.0) | 0.138 | 1 (1.8) |  | 1.000 |
|  | 10A | 46 (6.1) | 13 (4.1) | 0.192 |  |  |  |
|  | 15B | 32 (4.2) | 12 (3.8) | 0.734 | 4 (12.5) | 1 (8.3) | 1.000 |
|  | 22F | 27 (3.6) | 12 (3.8) | 0.867 |  |  |  |
|  | 33F | 12 (1.6) | 3 (0.9) | 0.594 |  |  |  |
|  | 20 | 9 (1.2) | 2 (0.6) | 0.617 |  |  |  |
|  | 12F | 8 (1.1) | 1 (0.3) | 0.395 |  |  |  |
|  | 9N | 2 (0.3) |  | 0.887 |  |  |  |
|  | 6C | 43 (5.7) | 11 (3.4) | 0.129 |  |  |  |
| NVT^b^ | | 332 (43.7) | 191 (59.9) | **<0.001** | 72 (21.7) | 49 (25.7) | 0.300 |
|  | 35B | 83 (10.9) | 40 (12.5) | 0.445 | 66 (79.5) | 35 (87.5) | 0.406 |
|  | 15A | 72 (9.5) | 31 (9.7) | 0.901 | 2 (2.8) |  | 0.089 |
|  | 23A | 35 (4.6) | 13 (4.1) | 0.700 |  |  |  |
|  | 15C | 25 (3.3) | 25 (7.8) | **0.001** | 2 (8.0) | 6 (24.0) | 0.247 |
|  | 34 | 24 (3.2) | 21 (6.6) | **0.010** |  |  |  |
|  | 24F | 12 (1.6) | 9 (2.8) | 0.268 |  | 8 (88.9) | **<0.001** |
|  | 37 | 10 (1.3) | 11 (3.4) | **0.021** |  |  |  |
|  | 23B | 10 (1.3) | 10 (3.1) | **0.043** |  |  |  |
|  | 31 | 6 (0.8) | 2 (0.6) | 0.916 |  |  |  |
|  | 24B | 5 (0.7) | 6 (1.9) | 0.135 |  |  |  |
|  | 28F | 4 (0.5) | 2 (0.6) | 0.806 |  |  |  |
|  | 38 | 4 (0.5) | 1 (0.3) | 0.983 |  |  |  |
|  | 7C | 2 (0.3) | 2 (0.6) | 0.728 |  |  |  |
|  | 13 | 2 (0.3) |  | 0.887 | 2 (100) |  | 1.000 |
|  | 21 | 2 (0.3) | 7 (2.2) | **0.005** |  |  |  |
|  | 22A | 1 (0.1) |  | 0.654 |  |  |  |
|  | 35F |  | 3 (0.9) | **0.041** |  |  |  |
|  | 6D |  | 2 (0.6) | 0.159 |  |  |  |
|  | 16F |  | 1 (0.3) | 0.654 |  |  |  |
|  | 18B |  | 1 (0.3) | 0.654 |  |  |  |
|  | NT^e^ | 35 (4.6) | 4 (1.3) | **0.012** |  |  |  |
| Total | | 760 | 319 |  | 130 (17.1) | 59 (18.5) | 0.584 |

^a^ Vaccine type: serotypes included in PCV13 or PPSV23 and serotype 6C; ^b^ Non-vaccine type: serotypes not included in PCV13 and PPSV23 or serotype 6C; ^c^ Rates in isolates; ^d^ Rates in the serotype; ^e^ Non-typeable
