# Supplementary material for: Bacteriological characteristics and changes of Streptococcus pneumoniae serotype 35B after vaccine implementation in Japan
Source: Epidemiol Infect. 2024 Oct 4;152:e114. doi: 10.1017/S0950268824001031 (PMC11450500; doi:10.1017/S0950268824001031)
Supplement: Miyazaki et al. supplementary material 4 — Miyazaki et al. supplementary material [file S0950268824001031sup004.docx]

Supplementary Table S4.　Serotype and penicillin susceptibility of *Streptococcus pneumoniae* isolates

|  |  | Isolates | | MIC^c^ ≥2 μg/mL | | | | | 0.12 μg/mL ≤ MIC^c^ ≤1 μg/mL | | | MIC^c^ ≤0.06 μg/mL | | |
| --- | --- | --- | --- | --- | --- | --- | --- | --- | --- | --- | --- | --- | --- | --- |
|  |  | 2014-  2017 | 2018-2022 | 2014-  2017 | 2018-  2022 | | *p* | | 2014-  2017 | 2018-  2022 | *p* | 2014-  2017 | 2018-  2022 | *p* |
|  |  | n | n | n (%^d^) | n (%^d^) | |  | | n (%^d^) | n (%^d^) |  | n (%^d^) | n (%^d^) |  |
| VT^a^ |  | 428 | 127 | 13 (3.0) | 7 (5.5) | | 0.297 | | 74 (17.3) | 15 (11.8) | 0.140 | 341 (79.7) | 105 (82.7) | 0.454 |
|  | 3 | 66 | 29 | 1 (1.5) |  | | 1.000 | | 2 (3.0) |  | 1.000 | 63 (95.5) | 29 (100) | 0.551 |
|  | 19A | 62 | 15 |  |  | |  | | 15 (24.2) | 3 (20.0) | 1.000 | 47 (75.8) | 12 (80.0) | 1.000 |
|  | 6B | 20 | 5 | 3 (15.0) | 2 (40.0) | | 0.252 | | 14 (70.0) | 1 (20.0) | 0.121 | 3 (15.0) | 2 (40.0) | 0.252 |
|  | 19F | 18 | 3 | 4 (22.2) |  | | 1.000 | | 12 (66.7) | 1 (33.3) | 0.531 | 2 (11.1) | 2 (66.7) | 0.080 |
|  | 7F | 10 | 1 |  |  | |  | |  |  |  | 10 (100) | 1 (100) | 1.000 |
|  | 6A | 5 | 1 | 4 (80.0) |  | | 0.333 | |  |  |  | 1 (20.0) | 1 (100) | 0.333 |
|  | 23F | 3 | 3 |  |  | |  | | 3 (100) | 2 (66.7) | 1.000 |  | 1 (33.3) | 1.000 |
|  | 1 | 3 |  |  |  | |  | |  |  |  | 3 (100) |  |  |
|  | 18C | 3 |  |  |  | |  | |  |  |  | 3 (100) |  |  |
|  | 9V | 1 |  |  |  | |  | |  |  |  | 1 (100) |  |  |
|  | 14 | 1 |  |  |  | |  | |  |  |  | 1 (100) |  |  |
|  | 11A/E | 57 | 16 | 1 (1.8) |  | | 1.000 | | 5 (8.8) |  | 0.579 | 51 (89.5) | 16 (100) | 0.328 |
|  | 10A | 46 | 13 |  |  | |  | | 3 (6.5) | 1 (7.7) | 1.000 | 43 (93.5) | 12 (92.3) | 1.000 |
|  | 15B | 32 | 12 |  | 1 (8.3) | | 0.273 | | 5 (15.6) | 3 (25.0) | 0.663 | 27 (84.4) | 8 (66.7) | 0.195 |
|  | 22F | 27 | 12 |  |  | |  | | 1 (3.7) |  | 1.000 | 26 (96.3) | 12 (100) | 1.000 |
|  | 33F | 12 | 3 |  |  | |  | |  |  |  | 12 (100) | 3 (100) | 1.000 |
|  | 20 | 9 | 2 |  |  | |  | |  |  |  | 9 (100) | 2 (100) | 1.000 |
|  | 12F | 8 | 1 |  |  | |  | | 1 (12.5) |  | 1.000 | 7 (87.5) | 1 (100) | 1.000 |
|  | 9N | 2 |  |  |  | |  | |  |  |  | 2 (100) |  |  |
|  | 6C | 43 | 11 |  | 4 (36.4) | | **0.001** | | 13 (30.2) | 4 (36.4) | 0.696 | 30 (69.8) | 3 (27.3) | **0.015** |
| NVT^b^ |  | 332 | 189 | 21 (6.3) | 20 (10.6) | | 0.083 | | 177 (53.3) | 72 (38.1) | **0.001** | 134 (40.4) | 97 (51.3) | **0.015** |
|  | 35B | 83 | 40 | 11 (13.3) | 10 (25.0) | | 0.105 | | 57 (68.1) | 25 (62.5) | 0.496 | 15 (18.1) | 5 (12.5) | 0.433 |
|  | 15A | 72 | 31 | 3 (4.2) | 8 (25.8) | | **0.003** | | 62 (86.1) | 22 (71.0) | 0.069 | 7 (9.7) | 1 (3.2) | 0.188 |
|  | 23A | 35 | 13 |  |  | |  | | 33 (94.3) | 13 (100) | 1.000 | 2 (5.7) |  | 1.000 |
|  | 15C | 25 | 25 |  |  | |  | | 4 (16.0) | 9 (36.0) | 0.107 | 21 (84.0) | 16 (64.0) | 0.107 |
|  | 34 | 24 | 21 |  |  | |  | | 1 (4.2) |  | 1.000 | 23 (95.8) | 21 (100) | 1.000 |
|  | 24F | 12 | 8 |  |  | |  | |  |  |  | 12 (100) | 8 (100) | 1.000 |
|  | 37 | 10 | 11 |  |  | |  | |  |  |  | 10 (100) | 11 (100) | 1.000 |
|  | 23B | 10 | 10 |  |  | |  | |  | 2 (20.0) | 0.474 | 10 (100) | 8 (80.0) | 0.474 |
|  | 31 | 6 | 2 |  |  | |  | |  |  |  | 6 (100) | 2 (100) | 1.000 |
|  | 24B | 5 | 6 |  |  | |  | |  |  |  | 5 (100) | 6 (100) | 1.000 |
|  | 28F | 4 | 2 |  |  | |  | |  |  |  | 4 (100) | 2 (100) | 1.000 |
|  | 38 | 4 | 1 |  |  | |  | |  |  |  | 4 (100) | 1 (100) | 1.000 |
|  | 7C | 2 | 2 |  |  | |  | |  |  |  | 2 (100) | 2 (100) | 1.000 |
|  | 13 | 2 |  |  |  | |  | | 1 (50.0) |  |  | 1 (50.0) |  |  |
|  | 21 | 2 | 7 |  |  | |  | |  |  |  | 2 (100) | 7 (100) | 1.000 |
|  | 22A | 1 |  |  | |  | |  |  |  |  | 1 (100) |  |  |
|  | 35F |  | 3 |  | |  | |  |  |  |  |  | 3 (100) |  |
|  | 6D |  | 2 |  | | 1 (50.0) | |  |  |  |  |  | 1 (50.0) |  |
|  | 16F |  | 1 |  | |  | |  |  |  |  |  | 1 (100) |  |
|  | 18B |  | 1 |  | |  | |  |  |  |  |  | 1 (100) |  |
|  | NT^e^ | 35 | 3 | 7 (20.0) | | 1 (33.3) | | 1.000 | 19 (54.3) | 1 (33.3) | 0.595 | 9 (25.7) | 1 (33.3) | 1.000 |
| Total |  | 760 | 316 | 34 (4.5) | | 27 (8.5) | | **0.009** | 251 (33.0) | 87 (27.5) | 0.077 | 475 (62.5) | 202 (63.9) | 0.660 |

^a^ Vaccine type: serotypes included in PCV13 or PPSV23 and serotype 6C; ^b^ Non-vaccine type: serotypes not included in PCV13 and PPSV23 or serotype 6C; ^c^ minimum inhibitory concentration (µg/mL); ^d^ Rates in the serotype; ^e^ Non-typeable
